# Morphological Characterization and Assessment of Genetic Variability, Character Association, and Divergence in Soybean Mutants

**DOI:** 10.1155/2014/968796

**Published:** 2014-08-12

**Authors:** M. A. Malek, Mohd Y. Rafii, Most. Shahida Sharmin Afroz, Ujjal Kumar Nath, M. Monjurul Alam Mondal

**Affiliations:** ^1^Bangladesh Institute of Nuclear Agriculture, Mymensingh 2202, Bangladesh; ^2^Institute of Tropical Agriculture, Universiti Putra Malaysia (UPM), 43400 Serdang, Selangor, Malaysia; ^3^Cotton Development Board, Farmgate, Dhaka 1215, Bangladesh; ^4^Bangladesh Agricultural University, Mymensingh 2202, Bangladesh

## Abstract

Genetic diversity is important for crop improvement. An experiment was conducted during 2011 to study genetic variability, character association, and genetic diversity among 27 soybean mutants and four mother genotypes. Analysis of variance revealed significant differences among the mutants and mothers for nine morphological traits. Eighteen mutants performed superiorly to their mothers in respect to seed yield and some morphological traits including yield attributes. Narrow differences between phenotypic and genotypic coefficients of variation (PCV and GCV) for most of the characters revealed less environmental influence on their expression. High values of heritability and genetic advance with high GCV for branch number, plant height, pod number, and seed weight can be considered as favorable attributes for soybean improvement through phenotypic selection and high expected genetic gain can be achieved. Pod and seed number and maturity period appeared to be the first order traits for higher yield and priority should be given in selection due to their strong associations and high magnitudes of direct effects on yield. Cluster analysis grouped 31 genotypes into five groups at the coefficient value of 235. The mutants/genotypes from cluster I and cluster II could be used for hybridization program with the mutants of clusters IV and V in order to develop high yielding mutant-derived soybean varieties for further improvement.

## 1. Introduction

Cultivated soybean [*Glycine max *(L.) Merr.], one of the major crops, is used for animal feed and human foods [1]. Unlike most of the vegetable proteins, soybean protein supplies all the essential amino acids, having cardio friendly oil which fulfills 30 percent of world vegetable oil requirement and also has many therapeutic components, namely, lactose-free fatty acids, antioxidants and folic acid, vitamin B complex, and isoflavones [2]. Due to the versatile nature of this crop, its contribution to industrial, agricultural, and medicinal sectors is significantly increasing. Rapid increase of population together with gradual reduction of cultivable land has posed greater challenges to human health in Bangladesh. As a result, the present diet pattern in Bangladesh is highly imbalanced with deficit consumption of both pulse and oils. In this circumstance, soybean can be the excellent source of balance diet to meet the nutritional deficiencies in Bangladesh. The average yield of soybean in Bangladesh is 1.64 tons per ha only compared to world average yield of 3.0 tons per ha [3]. Among the factors responsible for its lower yield in Bangladesh, the most important is the nonavailability of high yielding varieties.

In soybean, creation of genetic variation through hybridization is a tedious process due to small and fragile flowers, which make it very difficult to carry out the process of emasculation and injuring the parts of the flower and are prone to heavy flower shedding even under favorable conditions. These coupled with complete self-fertility impose limitations on the success of hybridization program [4]. As a result, mutation breeding appears to play an important role in creating genetic variability for improving this important crop.

Kharkwal and Shu [5] reported that induced mutation breeding is becoming more powerful and effective in breeding crop varieties to play a significant role for improving world food security in the coming years and decades. Induced mutations have generated a vast amount of genetic variability and are now widely used for the development of genes controlling important traits and understanding the functions and mechanisms of actions of these genes in plants [6]. Mutation breeding is now playing an important role in developing new genetic resources and breakage of unwanted linkages [7]. Using mutation breeding, genetic improvement of any yield attributes either qualitative or quantitative trait, has been successfully achieved in soybean [8–16] and also in other oil crops like rapeseed-mustard [17–19]. Furthermore, mutation breeding requires less time to develop crop cultivars as compared to the conventional breeding [20, 21]. The commercial utilization of approximately 3,000 mutant-induced and mutant-derived varieties strongly shows the contribution of mutation breeding to generating new germplasm for crop improvement [22].

The information as well as assessment of genetic variability in the existing germplasm of a particular crop is sought as prerequisite [23–25]. Furthermore, heritability of a plant trait is very important in determining the response to selection because it implies the extent of transmissibility of traits into next generations [26]. In addition, high genetic advance coupled with high heritability estimate offers the most effective condition for selection for a particular trait [27].

Increased seed yield is the ultimate goal of the breeders. But seed yield itself is a product of interaction of many component traits which influence yield directly or indirectly. So, it is important to see the contribution of each of the traits in order to give more attention to those having the highest influence on yield. Moreover, understanding the relationship between yield and its component traits is of great importance to a breeder for making the best use of these relationships in selecting desirable genotypes for yield improvement programs [28, 29]. As correlation alone cannot explain relationships among the characters, therefore the path coefficient analysis has been used in different crop species for complete determination of the impact of the independent variables on the dependent one and to find direct and indirect effects [30]. Therefore, to identify the traits which have significant effect on yield for potential use in selection, path analysis has been widely used in crop breeding program [31, 32].

This study investigated the morphological variability among 27 soybean mutants along with four mother varieties using quantitative morphological traits including yield attributes. For an effective breeding program for crop variety development through hybridization, the analysis of genetic diversity is one of the useful tools and plays a fundamental role in identification of parents [33, 34]. Moreover, better knowledge on genetic diversity could help to achieve long-term selection gain [35]. As a traditional method, morphological traits are used to assess genetic divergence and classify existing germplasm materials. However, this technique, a low level but powerful taxonomic tool, has been utilized for the preliminary grouping of germplasm prior to their characterization using more precise marker technologies. According to Din et al. [36] scientific classification of the plant still relies on morphological traits. Moreover, this technique is easier, cost effective, and easy to score and requires less time and finally it does not need any technical knowledge.

From four mother genotypes (Sohag, BARI Soybean-5, Bangladesh Soybean-4, and BAU-S/64), Bangladesh Institute of Nuclear Agriculture (BINA) developed 27 true breeding soybean mutants using gamma rays from the Co^60^ gamma cell. Among those mutants, 18 promising mutants showed better performance in respect to seed yield per ha along with other morphological traits including important yield attributes than the mother varieties/line. In this research, we evaluated the performances of those mutants along with mothers from January to June 2011 regarding morphological parameters and yield traits through the studies of genotypic and phenotypic variability, character association, and genetic diversity among these mutants and mothers which have not yet been studied. Such information will serve as a useful tool for establishing suitable breeding program for further soybean improvement.

## 2. Materials and Methods

### 2.1. Experimental Site

The experiment was carried out at the experimental field of Bangladesh Institute of Nuclear Agriculture (BINA), Mymensingh, during January to June 2011. Geographically, the place is located at about 24°75′ north latitude and 90°50′ east longitude. The soil of the experimental site is sandy loam having 0.06% nitrogen, 1.05% organic matter, 18.5 ppm available phosphorus, 0.28 meq% exchangeable potassium, 18 ppm sulphur, and 6.8 pH.

### 2.2. Plant Materials

Thirty-one soybean genotypes were used as the experimental materials. Among the genotypes, 27 were the true breeding M_6_ mutants and the other four were the mother genotypes, Sohag, Bangladesh Soybean-4 (BDS-4), BARI Soybean-5, and BAU-S/64, from which the mutants were evolved. The names of the 27 soybean mutants along with their respective mother genotype are listed in Table 1.

### 2.3. Experimental Design and Setting the Experiment

The experiment was laid out in a randomized complete block design with three replicates. Block-to-block and plot-to-plot distances were maintained as 1.25 and 0.75 m, respectively, with a plot size of 4.0 m × 3.6 m and line-to-line distance of 30 cm. Seeds were sown on 26 December 2010. Each entry was grown in 12 rows keeping plant-to-plant distance of 8–10 cm in rows.

### 2.4. Intercultural Operations

Urea, triple super phosphate, muriate of potash, and gypsum were used as basal dose during final land preparation at 40, 150, 100, and 110 kg ha^−1^, respectively.* Rhizobium* inoculum for soybean was used at 25 g per kg seeds. Intercultural operations like weeding, thinning, application of pesticide, and so forth were done as recommended and when necessitated for proper growth and development of plants in each plot. Harvesting was done depending upon the maturity of the plants in each plot.

### 2.5. Data Collection

Data on plant height, number of primary branches and pods per plant, number of seeds per pod, and seed yield per plant were taken from 10 randomly selected competitive plants from each plot. Plants of each plot were harvested when the plants and pods of each plot turned into yellowish brown colour and almost all the leaves shed. Plot seed yield was taken from the eight middle rows avoiding border effects and plot seed yield was converted into kg per ha (Table 2).

### 2.6. Statistical Analyses

Analysis of variance (ANOVA) and least significant difference (LSD) were computed for all traits using SAS 9.1 for identification of significant difference between progenies. Genetic parameters were estimated by the formula given by Burton [37], Burton and Vane [38], and Johnson et al. [39]. These parameters include the following:

- *σ* ^2^_G_ (an estimate of genotypic variance) = (MS_G_ − MS_E_)/*r*, where MS_G_ is an estimate of mean square of tested accession, MS_E_ is an estimate of mean square of error, and *r* refers to the number of replications;
- MS_E_ is an estimate of *σ* ^2^_E_;
- *σ* ^2^_P_ (an estimate of phenotypic variance) = *σ* ^2^_G_ (genotypic component of variance) + *σ* ^2^_E_;
- PCV (phenotypic coefficient of variation) =$\sqrt{{\sigma^{2}}_{\text{P}}}/\bar{X}\times 100$, where *σ* ^2^_P_ is the phenotypic component of variance and $\bar{X}$ is the mean of the trait;
- GCV (genotypic coefficient of variation) =$\sqrt{{\sigma^{2}}_{\text{G}}}/\bar{X}\times 100$, where *σ* ^2^_G_ is the genotypic component of variance and $\bar{X}$ is the mean of the trait;
- *h* ^2^_B_ (an estimate of broad sense heritability) = *σ* ^2^_G_/*σ* ^2^_p_, where *σ* ^2^_G_ is the genotypic component of variance and *σ* ^2^_P_ is the phenotypic component of variance;
- GA (genetic advance) is taken as percent of the mean assuming selection of the superior 5% of the accessions;
- GA (as % of the mean) = $K\times \sqrt{{\sigma^{2}}_{\text{P}}}/\bar{X}\times {h_{\text{B}}}^{2}\times 100$, where *K* (the standardized selection intensity) = 2.06 (at 5% selection intensity), *σ* ^2^_P_ is the phenotypic component of variance, *h* ^2^_B_ is the heritability in broad sense, and $\bar{X}$ refers to the mean of the trait being evaluated.

Genotypic and phenotypic correlation coefficients for different characters were calculated in all possible combinations following the formula given by Miller et al. [40]. Path coefficient analysis was done following Dewey and Lu [24], also quoted by Singh and Chaudhury [41] and Dabholkar [42]. For cluster analysis, data were analyzed to determine Euclidean distance based on paired group method to determine dissimilar groups of the mutants. Two-dimensional principal component analysis (PCA) graph was constructed using PAST-multivariate software.

## 3. Results

### 3.1. Variability and Genetic Parameters among the Mutants

ANOVA showed that mean squares due to genotypes were highly significant (*P* ≤ 0.01) for all the nine characters like days to flowering and maturity, plant height, number of branches and pods per plant, seeds per pod, 100-seed weight, seed yield per plant, and seed yield per ha (Table 3). These results revealed highly significant genotypic variations among the genotypes for all these traits. Phenotypic and genotypic coefficients of variation (PCV and GCV), broad sense heritability, and genetic advance were calculated for all the characters (Table 4). The highest PCV and GCV were observed for branches per plant (38.11 and 35.03%, resp.) and the lowest PCV and GCV were recorded for days to maturity (7.22 and 6.35%, resp.). The PCV and GCV of plant height (19.16 and 17.91%), pods per plant (18.16 and 16.59%), 100-seed weight (16.97 and 16.43%), and seed yield per ha (14.06 and 12.61%) were higher compared to days to flowering (8.36 and 7.56%) and days to maturity (7.22 and 6.35%). Results also showed narrow differences between PCV and GCV for most of the traits. All the characters exhibited high heritability which ranged from 77.40% in days to maturity to 93.73% in 100-seed weight. Among the traits, only days to maturity had relatively low heritability. The genetic advance as percent of mean (GA%) ranged from 11.50% in days to maturity to 66.33% in branches per plant. Among the traits, number of branches per plant, plant height, 100-seed weight, and pods per plant exhibited higher percentages of genetic advance.

### 3.2. Performance of the Mutants and Mothers

Mean performances of the mutants along with the mothers for different morphological traits are shown in Table 5. The shortest time required to flowering and maturity (58 and 116 days) was observed in mutant SBM-15 closely followed by SBM-16 (59 and 116 days) and the longest (80 and 150 days) was required in BAU-S/64. Results also showed that some of the mutants required significantly lower flowering and maturity period than their respective mothers. Most of the mutants from Sohag produced significantly lower plant height and lower number of branches per plant, but 11 mutants produced significantly higher number of pods per plant and seed yield (per plant and ha), and only two mutants (SBM-08 and SBM-10) gave significantly higher seed weight than Sohag. On the other hand, the mutants from BARI Soybean-5 and BDS-4, most of the mutants produced significantly taller plant than their respective mothers and statistically similar number of branches and pods per plant. Among four mutants, three (SBM-11, SBM-13, and SBM-14) produced significantly higher seed yield per plant and per ha than mother variety Bangladesh Soybean-4. Among nine mutants of BARI Soybean-5, six produced significantly higher 100-seed weight as well as seed yield per plant and per ha than mother. Among the two mutants of BAU-S/64, SBM-27 produced significantly higher 100-seed weight as well as seed yield per plant and per ha than mother.

### 3.3. Association among the Traits

Genetic and phenotypic correlations were calculated (Table 6) followed by path coefficient analysis to partition the correlation coefficients of traits with yield per plant into direct and indirect effects (Table 7). Genotypic correlations were found to be higher than the phenotypic correlations in most of the cases. Except for 100-seed weight, all other traits showed significant positive correlations with seed yield per plant and seed yield per ha both at genotypic and at phenotypic levels. Besides these, 100-seed weight also showed significant negative correlations with all other traits except seed yield per plant. Plant height showed highly significant positive correlation with branches per plant and both traits also showed significant positive correlations with most of the other traits. Days to flowering and days to maturity were positively and highly correlated and both traits showed significant positive correlation with plant height, branches per plant, and pods per plant and no significant correlation with seeds per pod.

Results of path coefficient analysis based on genotypic correlation of all the morphological traits indicated that, among the traits, seeds per pod had the highest direct positive effect (1.450) on seed yield per plant followed by 100-seed weight (1.350), days to maturity (1.184), and pods per plant (0.659). Days to flowering, plant height, and branches per plant having significant positive correlation with yield (0.646∗∗, 0.589∗∗, and 0.387∗, resp.) contributed mainly towards seed yield via days to maturity (1.102, 0.736, and 0.459, resp.), pods per plant (0.253, 0.543, and 0.528, resp.), and seeds per pod (0.405, 1.050, and 1.150, resp.) with negative direct effects (−0.646, −0.258, and −0.285, resp.). Pods per plant and seeds per pod contributed negatively towards seed yield via 100-seed weight (−1.040 and −1.168, resp.).

### 3.4. Cluster Analysis

**Figure 1:**
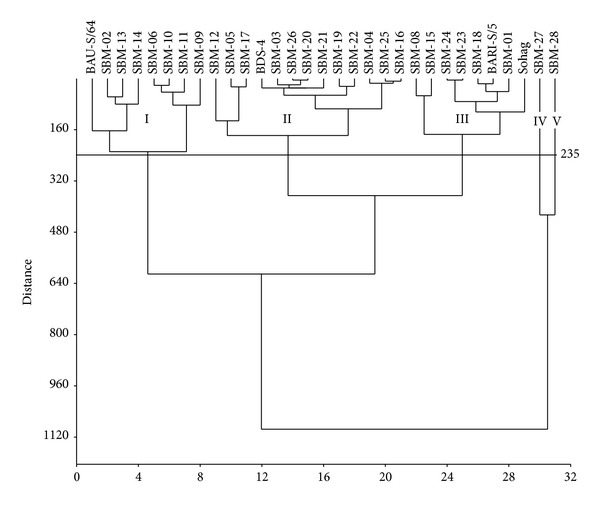
Dendrogram showing relationship among 31 soybean genotypes using nine phenological and morphological characters, seed yield, and yield traits.

Cluster analysis using all the nine morphological traits grouped the 31 accessions into five major groups at the genetic distance of 235.0 (Table 8, Figure 1). It was also found that, among the five clusters, cluster II was the largest and consisted of 13 genotypes (12 mutants and BDS-4) and the second largest group was the clusters I and III, and each consisted of eight genotypes. The smallest group was clusters IV and V, and each cluster contained only one mutant. Mean values of nine different traits for six groups among 31 soybean genotypes are presented in Table 9. Results showed that, among the five clusters, IV had the highest average means for all the traits except seeds per pod followed by clusters V and I. On the contrary, cluster III revealed the lowest means for all the traits.

### 3.5. Principal Component Analysis (PCA)

**Figure 2:**
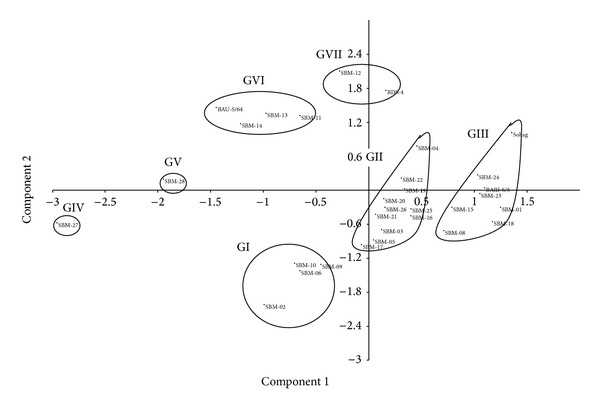
Two-dimensional plot of PCA showing relationships among 31 soybean genotypes using morphological and yield related traits. Note: BDS-4: Bangladesh Soybean-4; BARI-S/5: BARI Soybean-5.

A two-dimensional principal component analysis was performed using all the morphological traits. The cluster analysis was mostly confirmed by the PCA analysis. Two distant mutants such as SBM-27 and SBM-28formed their individual cluster/group alone both in cluster (clusters IV and V) and in PCA analyses (GIV and GV) (Figures 1 and 2, resp.). Four mutants, namely, SBM-02, SBM-06, SBM-09, and SBM-10, formed one group (GI), and BAU-S/64 formed another group (GVI) with mutants SBM-11, SBM-13, and SBM-14 though these seven mutants and BAU-S/64 together formed single cluster (cluster I) in cluster analysis. BDS-4 and SBM-12 formed one group (GVII), and 11 mutants formed another group (GII), though all these 12 mutants and Bangladesh Soybean-4 together formed single cluster (cluster II) in cluster analysis. Sohag formed group with BARI Soybean-5 with other six mutants both in cluster (cluster III) and in PCA analyses (GIII).

According to PCA, the first four principal components accounted for about 99.999% of total variation for all the morphological traits and exhibited high correlation among the traits analyzed.

## 4. Discussion

All the nine morphological traits showed highly significant (*P* ≤ 0.01) variations indicating the presence of sufficient amount of genetic variability among the mutants for all the studied traits. In soybean genotypes, significant variations have also been reported earlier by other researchers for various morphological traits [43–46]. Narrow differences between PCV and GCV for most of the traits indicate less influence of environmental factors on the expression of these traits and the chance of high selection gain. The heritability estimates help the breeders in selection based on the basis of phenotypic performance. Heritability and GA together with GCV could provide the best image of the amount of advancement to be expected through phenotypic selection [39]. So, high values of heritability and GA (%) along with high GCV for the characters like plant height, number of branches and pods per plant, and 100-seed weight can be considered as favorable morphological traits for soybean improvement through effective phenotypic selection of these traits and high expected genetic gain from selection for these characters can be achieved. This also indicates that these characters are under the control of additive gene action and would respond very well to continuous selection [47]. However, high heritability and GA (%) along with low GCV for the rest of the traits like days to flowering and maturity, seeds per pod, and seed yield per plant and per ha indicated that expression of these traits is under the involvement of nonadditive gene action and phenotypic selection of these traits might not be effective.

In plant breeding, creation of new plant type with improvement characters leading to producing high yield is the main objective. In soybean, the important yield attributes are the number of pods per plant, seeds per pod, and seed weight, which determine the seed yield.

In the present study, it was observed that, among the 27 mutants, 18 performed superiorly to their respective mothers in respect to seed yield per ha along with some other morphological traits including yield attributes like number of pods per plant and number of seeds per pod along with higher 100-seed weight, which contributed to the mutants in producing higher seed yield. These results are in agreement with the results of Tulmann et al. [48], Kundi et al. [49], Hussain et al. [50], and Ahire et al. [51], who reported improvement in yield attributes in soybean mutants as a consequence of mutagenesis.

Generally, estimates of genotypic correlation coefficients were found to be higher than their respective phenotypic correlation coefficients (Table 6), which are in agreement with the results of Weber and Moorthy [52] and Anand and Torrie [53]. Weber and Moorthy [52] also explained their result of low phenotypic correlation due to the masking or modifying effect of environment on the genetic association among the traits. The genotypic correlations of pods per plant and seeds/pod with days to flowering and maturity were positive, and the correlation between these two traits was very high (0.864∗∗) indicating that late maturing genotypes have more number of pods per plant and seeds per pod and consequently give higher seed yield. Seed weight always showed negative correlations with other desirable yield traits [54, 55] which indicates that the increase in one trait would result in the reduction of the other; that is, simultaneous increase or decrease of both traits would be difficult. The strong negative correlation of seed weight with other yield traits indicated that it would be very difficult to identify a soybean genotype having higher seed weight simultaneously with higher number of pods per plant and seeds per pod; rather an increase in one trait would result in the reduction of the others. Significant positive correlations of days to flowering and maturity, plant height, branches and pods per plant, seeds per pod, and seed weight with seed yield (Table 6) indicate that in selecting high yielding genotypes these characters should be given more emphasis as the best selection criteria. These results also are in agreement with the results reported by others in soybean [30, 45, 53, 55–58]. Machikowa et al. [57] also reported that days to flowering and maturity were highly and positively correlated with yield components in soybean. Highly significant and positive correlation between seed yield per plant and yield per ha indicates that in soybean individual plant yield contributed significantly towards yield per unit area. Significant positive correlation of plant height with days to maturity indicates that genotypes with taller plants tend to longer maturity period.

In soybean, positive direct effects of number of pods per plant [54, 55, 59] and days to maturity [30] on seed yield were also reported and showed similarity with the present results. The direct effect of 100-seed weight on seed yield was also positive (1.350) having high negative indirect effect through seeds per pod (−1.258) and pods per plant (−0.521). Therefore, the negative indirect effects of 100-seed weight with these traits will be a problem in combining these important characters for high seed yield. Among the traits, indirect effects through pods per plant, seeds per pod, and days to maturity were found to be important and these results agreed partially with the findings of Iqbal et al. [60] and Machikowa and Laosuwan [55], who reported high indirect effects through pods per plant and maturity period. Therefore, days to maturity is also suggested to be an important selection criterion in soybean for seed yield. Faisal et al. [30] and Harer and Deshmukh [61] also reported similar results and suggested greater emphasis on longer duration during selection. Present results also suggest that soybean yield could be increased through the selection of higher number of pods per plant with higher number of seeds per pod and longer maturity period. Therefore, in soybean, pod number per plant and seeds per pod and days to maturity can be considered as the major and effective characters influencing the seed yield in soybean. Both the correlation and path analyses indicate that pod number per plant and seeds per pod and days to maturity appeared to be the first order yield components and priority should be given during selection due to having strong associations as well as high direct effects on seed yield.

Clustering analysis based on nine morphological traits grouped 31 soybean genotypes into five different clusters and indicates that 31 soybean genotypes exhibited notable genetic divergence in terms of morphological traits. Therefore, classification in this study based on morphological traits is in agreement with previous report. Formation of different number of clusters using morphological characters in diverse soybean genotypes was also reported [45, 62, 63]. The dendrogram tends to group some of the mutants with similar morphological traits into the same cluster. Similar results were also reported in soybean and other crops by Cui et al. [62], Yu et al. [64], Iqbal et al. [63], Abdullah et al. [65], Latif et al. [66], and Rafii et al. [67].

Results revealed that, among 13 mutants from Sohag and nine mutants from BARI Soybean-5, only three (SBM-08, SBM-10, and SBM-24) from Sohag and only three (SBM-15, SBM-18,and SBM-23) from BARI Soybean-5 formed cluster with mother varieties Sohag and BARI Soybean-5, respectively, and others formed distinct clusters other than the mother genotypes. Similarly among four mutants from Bangladesh Soybean-4, only one (SBM-12) formed cluster with mother, and both mutants SBM-27 and SBM-28 from BAU-S/64 formed two individual clusters. Present results confirm that induced mutations are contributing significantly to creating genetic variations in crop plants. The first four principal components accounted for 99.999% of the total variation. Cluster analysis using dendrogram and PCA following two-dimensional method played complementary role to each other with little inconsistencies in respect of number of genotypes in cluster formation. To obtain greater heterosis, genotypes having distant clusters could be used as parents for hybridization program. Dendrogram and two-dimensional PCA graph clearly indicated that mutants SBM-27 and SBM-28 made two individual groups (clusters IV and V, resp.) and were far away from the other three clusters. Therefore, the mutants from cluster I and cluster II could be used for hybridization program with the mutants of clusters IV (SBM-27) and V (SBM-28) in order to develop high yielding mutant-derived soybean varieties.

## 5. Conclusion

In plant breeding, generation of new genotypes from the existing ones with improvement in plant traits is the main objective. The present study revealed the presence of high levels of variations for nine different morphological traits including yield attributes and seed yield among the newly developed 27 mutants along with four mother genotypes of soybean. These mutants could be served as raw materials for further genetic improvement of different characters of the soybean. Among the nine traits, plant height, number of branches and pods per plant, and 100-seed weight exhibited high values of genotypic coefficient of variation, broad sense heritability, and genetic advance. Therefore, these traits can be considered as favorable attributes for soybean improvement through effective phenotypic selection and high expected genetic gain can be achieved for these characters. Most of the traits showed positive correlations between each other, which will assist in the combined improvement of these traits by selecting only highly heritable and easily measurable phenotypic traits. In addition, both the correlation and path coefficient analyses indicated that pod number per plant and seeds per pod and days to maturity appeared to be the first order traits for higher seed yield in soybean and priority should be given in selection due to strong associations as well as high magnitudes of direct effects on seed yield. Cluster analysis using all the nine different traits grouped 27 soybean mutants and four mother genotypes into five main clusters. These results also confirm that not only the geographical background, but also induced mutations significantly contribute to creating genetic variations. The first four principal components accounted for about 99.996% of total variation for all the morphological traits. This study indicated the presence of high levels of genetic diversity among the mutants for evaluated characters.

## Figures and Tables

**Table 1:** List of 27 soybean mutants with their mother varieties/line.

Name of the mutant	Mother variety/line	Name of the mutant	Mother variety/line
SBM-01	Sohag	SBM-18	BARI Soybean-5
SBM-02	Sohag	SBM-19	BARI Soybean-5
SBM-03	Sohag	SBM-20	BARI Soybean-5
SBM-04	Sohag	SBM-21	BARI Soybean-5
SBM-05	Sohag	SBM-22	BARI Soybean-5
SBM-06	Sohag	SBM-23	BARI Soybean-5
SBM-08	Sohag	SBM-24	Sohag
SBM-09	Sohag	SBM-25	Sohag
SBM-10	Sohag	SBM-26	Sohag
SBM-11	BDS-4	SBM-27	BAU S/64
SBM-12	BDS-4	SBM-28	BAU S/64
SBM-13	BDS-4	Sohag	Mother variety
SBM-14	BDS-4	BARI Soybean-5	Mother variety
SBM-15	BDS-4	BDS-4	Mother variety
SBM-16	BDS-4	BAU S/64	Mother line
SBM-17	BARI Soybean-5		

Note: BDS-4: Bangladesh Soybean-4.

**Table 2:** List of different traits and their description of measurement.

Serial number	Traits	Method of measurement
1	Days to flowering	The number of days from sowing to flowering of 50% plants
2	Days to maturity	The number of days from sowing until approximately 90% pod turned into brownish colour
3	Plant height (cm)	The height from the base of the plant to the tip of last leaf
4	Branches per plant (number)	Total number of pod bearing primary branches in a plant
5	Pods per plant (number)	Total number of pods with seed in a plant
6	Seeds per pod (number)	Total number of seeds in a pod
7	100-seed wt (g)	One hundred seeds randomly counted and then weighed
8	Seed yield per plant (g)	Weighing the total number of seeds produced in a plant
9	Seed yield (kg per ha)	Weighing the seeds produced in a plot and then converted into kg per ha

**Table 3:** Mean square values for nine different phenological and morphological characters, yield attributes, and seed yield among 31 soybean genotypes.

Sources of variation	DF	Days to flowering	Days to maturity	Plant height (cm)	Branches per plant (number)	Pods per plant (number)	Seeds per pod (number)	100-seed wt (gm)	Seed yield per plant (g)	Seed yield (kg per ha)
Replication	2	3.12	33.07	97.49	0.047	10.87	0.001	0.128	0.615	5902.91
Genotypes	30	75.06∗∗	201.2∗∗	439.08∗∗	4.974∗∗	203.88∗∗	0.153∗∗	11.735∗∗	4.082∗∗	535273∗∗
Error	60	5.24	17.84	21.09	0.289	12.70	0.011	0.256	0.326	40219

**Significant at 1% level of probability.

**Table 4:** Estimation of genetic parameters of nine different phenological and morphological characters, yield attributes, and seed yield among 31 soybean genotypes.

Characters	Genotypic variance	Phenotypic variance	Grand mean	Heritability (%)	GCV (%)	PCV (%)	GA (%)
Days to flowering	23.27	28.51	63.84	81.62	7.56	8.36	14.06
Days to maturity	61.11	78.95	123.15	77.40	6.35	7.22	11.50
Plant height (cm)	139.33	159.42	65.91	87.74	17.91	19.16	36.63
Branches per plant (number)	1.564	1.851	3.57	84.50	35.03	38.11	66.33
Pods per plant (number)	63.73	76.43	48.13	83.38	16.59	18.16	31.20
Seeds per pod (number)	0.047	0.058	1.96	81.03	11.06	12.29	20.51
100-seed weight (g)	3.83	4.08	11.91	93.73	16.43	16.97	32.76
Seed yield per plant (g)	1.252	1.578	9.50	79.34	11.78	13.51	22.08
Seed yield (kg per ha)	165018	205237	3221	80.40	12.61	14.06	23.29

**Table 5:** Mean performances of 27 soybean mutants and four mother varieties for nine different phenological and morphological characters, yield attributes, and seed yield.

Genotypes	DF	DM	Plant height (cm)	Branches per plant (number)	Pods per plant (number)	Seeds per pod (number)	100-seed wt (g)	Seed yield per plant (g)	Seed yield per ha (kg)
SBM-01	64	122	53	2.46	40	1.83	12.5	8.7	2675
SBM-02	62	120	57	2.80	45	2.00	13.0	10.6	3663
SBM-03	64	124	58	2.70	42	2.00	12.2	9.2	3126
SBM-04	64	126	71	4.60	45	1.83	11.7	9.1	3015
SBM-05	60	120	57	2.53	45	1.73	12.7	9.4	3202
SBM-06	64	120	58	2.70	48	1.96	13.4	11.1	3498
SBM-08	60	116	54	2.60	41	1.80	13.8	8.8	2913
SBM-09	64	120	54	4.46	51	2.10	12.5	10.1	3418
SBM-10	64	122	61	3.43	44	2.00	14.3	10.1	3518
SBM-24	60	118	58	2.63	47	1.80	11.9	8.3	2772
SBM-25	62	120	60	2.80	43	1.70	12.3	9.0	3017
SBM-26	61	120	63	2.90	45	1.80	11.8	9.4	3107
Sohag	66	125	65	5.26	38	1.86	12.8	8.2	2627
SBM-11	66	122	81	6.10	65	2.33	7.6	9.7	3479
SBM-12	66	122	86	5.60	64	2.36	7.7	9.4	3342
SBM-13	62	120	87	5.50	65	2.53	7.9	10.3	3619
SBM-14	62	121	87	6.26	64	2.40	7.7	10.8	3715
BDS-4	68	128	76	5.76	61	2.30	7.8	8.9	3127
SBM-15	58	116	59	2.13	43	1.80	11.9	8.3	2860
SBM-16	59	116	58	3.26	46	1.80	13.4	8.7	3012
SBM-17	60	118	55	2.80	51	1.76	13.7	9.2	3228
SBM-18	61	118	53	2.83	36	1.80	13.1	8.0	2709
SBM-19	62	120	65	2.40	44	2.00	11.6	9.0	3059
SBM-20	62	119	65	2.10	45	1.80	12.8	9.0	3111
SBM-21	60	118	66	2.30	42	2.03	12.4	9.2	3142
SBM-22	61	122	67	2.60	45	1.80	13.2	9.3	3083
SBM-23	60	120	57	3.00	42	1.76	13.2	8.8	2772
BARI-5	66	126	54	2.60	41	1.96	11.4	8.2	2721
SBM-27	76	145	85	4.80	55	2.06	13.2	13.6	4459
SBM-28	74	143	82	4.40	55	1.90	13.4	11.6	4032
BAU-S/64	80	150	90	4.30	53	2.00	12.4	10.8	3824

LSD_0.05_	3.74	6.90	6.55	0.49	5.82	0.24	0.83	0.78	284
SE (±)	0.90	1.47	2.16	0.24	1.48	0.04	0.36	0.21	76.6
SD	5.00	8.19	12.03	1.31	8.24	0.22	1.98	1.18	426
CV%	3.59	3.43	6.09	8.37	7.41	7.33	4.25	6.30	7.40

Note: BARI-S/5: BARI Soybean-5; BDS-4: Bangladesh Soybean-4.

**Table 6:** Genotypic (G) and phenotypic (P) correlation coefficients among nine morphological traits in 31 soybean genotypes.

Characters		Days to maturity	Plant height	Branches per plant (number)	Pods per plant (number)	Seeds per pod (number)	100-seed wt (g)	Seed yield per plant (g)	Seed yield (kg per ha)
Days to flowering	G	0.931∗∗	0.659∗∗	0.494∗∗	0.385∗	0.279	−0.117	0.646∗∗	0.627∗∗
	P	0.966∗∗	0.646∗∗	0.485∗∗	0.381∗	0.301	−0.090	0.620∗∗	0.627∗∗
Days to maturity	G		0.622∗∗	0.388∗	0.286	0.119	−0.004	0.667∗∗	0.629∗∗
	P		0.611∗∗	0.381∗	0.290	0.158	0.032	0.634∗∗	0.626∗∗
Plant height	G			0.776∗∗	0.824∗∗	0.725∗∗	−0.621∗∗	0.589∗∗	0.677∗∗
	P			0.771∗∗	0.805∗∗	0.696∗∗	−0.615∗∗	0.570∗∗	0.668∗∗
Branches per plant (number)	G				0.801∗∗	0.796∗∗	−0.705∗∗	0.387∗	0.457∗∗
	P				0.796∗∗	0.763∗∗	−0.700∗∗	0.380∗	0.458∗∗
Pods per plant (number)	G					0.864∗∗	−0.774∗∗	0.518∗∗	0.640∗∗
	P					0.821∗∗	−0.763∗∗	0.508∗∗	0.633∗∗
Seeds per pod (number)	G						−0.867∗∗	0.398∗	0.509∗∗
	P						−0.818∗∗	0.378∗	0.484∗∗
100-seed wt (g)	G							0.012	−0.129
	P							0.004	−0.120
Yield per plant (g)	G								0.986∗∗
	P								0.962∗∗

∗∗ and ∗ indicate significance at 1% and 5% level of probability, respectively.

**Table 7:** Partitioning of genotypic correlations into direct (bold) and indirect effects of eight morphological traits in 31 soybean genotypes by path analysis.

Items	Days to flowering	Days to maturity	Plant height	Branch per plant	Pods per plant	Seeds per pod	100-seed wt (gm)	Yield per plant
Days to flowering	**−0.646**	1.102	**−**0.170	**−**0.141	0.253	0.405	**−**0.157	0.646∗∗
Days to maturity	−0.601	**1.184**	**−**0.161	**−**0.111	0.188	0.173	**−**0.005	0.667∗∗
Plant height (cm)	−0.425	0.736	**−0.258**	**−**0.221	0.543	1.050	**−**0.836	0.589∗∗
Branches per plant (number)	−0.318	0.459	−0.201	**−0.285**	0.528	1.150	**−**0.949	0.387∗
Pods per plant (number)	−0.248	0.338	−0.213	**−**0.228	**0.659**	1.250	**−**1.040	0.518∗∗
Seeds per pod (number)	−0.180	0.141	−0.187	**−**0.227	0.569	**1.450**	**−**1.168	0.398∗
100-seed weight (g)	0.076	**−**0.0086	0.161	0.201	**−**0.510	**−**1.258	**1.350**	0.012

Bold figures indicate the direct effects.

Residual effect = −0.0446.

∗ and ∗∗ indicate significant at 1% and 5% level of probability, respectively.

**Table 8:** Groups of 27 soybean mutants and four mother varieties according to cluster analysis from nine phenological and morphological characters, yield attributes, and seed yield.

Cluster number	Number of genotypes	Percent	Genotypes
I	8	25.8	BAU-S/64, SBM-02, SBM-13, SBM-14, SBM-06, SBM-10, SBM-11, SBM-09
II	13	42.0	SBM-12, SBM-05, SBM-17, BDS-4, SBM-03, SBM-26, SBM-20, SBM-21, SBM-19, SBM-22, SBM-04, SBM-25, SBM-16
III	8	25.8	SBM-08, SBM-15, SBM-24, SBM-23, SBM-18, BARI-S/5, SBM-01, Sohag
IV	1	3.2	SBM-27
V	1	3.2	SBM-28

Note: BARI-S/5: BARI Soybean-5; BDS-4: Bangladesh Soybean-4.

**Table 9:** Mean values of nine different phenological and morphological characters, yield attributes, and seed yield for five groups revealed by cluster analysis among 31 soybean genotypes.

Characters	I	II	III	IV	V
Days to flowering	65.5	62.23	61.88	76.00	74.00
Days to maturity	124.38	121.00	120.13	145.00	143.00
Plant height (cm)	71.88	65.15	56.63	85.00	82.00
Branches per plant (number)	4.44	3.26	2.94	4.80	4.40
Pods per plant (number)	54.38	47.54	41.00	55.00	55.00
Seeds per pod (number)	2.17	1.92	1.83	2.06	1.90
100-seed weight (g)	11.10	11.79	12.58	13.20	13.40
Seed yield per plant (g)	10.44	9.14	8.41	13.60	11.60
Seed yield (kg per ha)	3592	3121	2756	4459	4032
